# The impact of IVF patients’ characteristics on their satisfaction and quality-of-life with overseas treatment: A mixed methods approach

**DOI:** 10.1097/MD.0000000000038682

**Published:** 2024-07-19

**Authors:** Naskhanym Chausheva, Macide Artac Ozdal

**Affiliations:** a Department of Health Management, Faculty of Health Sciences, European University of Lefke, TRNC-10 Mersin.

**Keywords:** in vitro fertilization (IVF), patient satisfaction, quality-of-life

## Abstract

Recent advances in infertility therapy, such as hormone medication and in vitro fertilization (IVF), have led to an increase in the demand for IVF. North Cyprus is a new medical tourist destination, and this study aimed to discover influential demographic predictors of IVF patients’ satisfaction and quality-of-life (QoL) after receiving reproductive IVF services. Two questionnaires on IVF patient satisfaction and QoL were administered to 101 patients who received services in selected IVF clinics. Parametric and nonparametric tests were used for statistical analysis. The results showed that the mean satisfaction level with IVF service introduction and doctor professionalism increased with age, and a maximum satisfaction level was found in older patients. Doctor professionalism was another significant factor for greater satisfaction in older patients than in young patients who underwent IVF treatment. Satisfaction with IVF services was reduced by increasing education levels. IVF services must be managed and provided based on the needs of patients from different demographic backgrounds and efforts must be made to improve satisfaction with fertility services.

## 1. Introduction

The vast majority of couples become pregnant when they expect to or even earlier if they are trying to conceive; however, 9% of all couples of childbearing age experience accidental childlessness.^[1]^ The World Health Organization classifies infertility as an illness, a serious health issue, and a social concern. Stigmatization due to infertility is widespread, and similar emotional responses have been recorded in various cultures.^[2]^ Many infertile couples have become biological parents using reproductive technology, which has improved the efficacy of infertility procedures. Recent advances in infertility therapy, such as hormone medications and in vitro fertilization (IVF), have led to an increase in the demand for infertility services^[3,4]^ and have promoted the use of medical techniques to treat infertility.^[5]^ Having a child is viewed as a perfectly reasonable desire by many societies worldwide.^[6]^ An increasing number of people are opting to seek medical treatment in other countries, a phenomenon known as medical tourism. Numerous websites are devoted to assisting individuals in their quest for medical facilities overseas that carry out their desired treatments, such as facelifts or reproductive procedures.^[6]^ Knowledge and tools for assisted reproduction are now widely available around the globe, prompting varied responses from governments, religious institutions, and public opinion.^[7,8]^

Traveling to another country for reproductive healthcare has become the focus of a new type of global market – the reproductive industry, frequently referred to as “reproductive tourism.”^[9]^ Many different factors motivate people to travel internationally in search of IVF treatment and other forms of assisted reproduction. The literature highlights factors such as cost differences between the homeland and the country of destination, ease of access in the home country (e.g. waiting times), a nonflexible legal system, and religious, governmental, and sociocultural issues regarding the availability of specific healthcare, as well as demographic variations (e.g. longer life expectancy) and personal standards. These factors can all affect the delivery of specific health services.^[10,11]^ The availability of new healthcare technology environments, exceptionally skilled clinical staff, facilities of top standards (in relation to patient services and facilities), certification of the care delivered, and an advantageous and forward-moving funding culture are all components that distinguish locations in their ability to attract larger numbers of medical tourists seeking assisted reproductive care.^[12]^

Despite the growing popularity of medical tourism, particularly for IVF procedures, the majority of studies have adopted a broad European Union-centric approach, with only a few studies attempting to research medical tourism and, specifically, IVF in North Cyprus.^[13,14]^ North Cyprus offers high success rates for IVF at costs significantly lower than Europe and the US, attracting many international medical tourists due to its liberal regulations.^[15]^ In the most recent research on reproductive travel, these trips have yet to be characterized. While this mode of travel has been considered, current perspectives and data on medical tourism have become more complicated.

There is a scarcity of studies on the perspectives of infertile patients in medical and patient-centered care. The technical competence and readiness of the staff to conduct examinations and administer treatments are examples of what constitutes “medical care.” Providing routine psychological support is a type of patient-centered care delivered by all staff members. Couples struggling with infertility should have access to care that is not only safe and reliable but also tailored to the needs of each individual patient.^[16,17]^ Patient perspectives and requirements are increasingly viewed as crucial determinants of healthcare quality.^[18,19]^ However, in fertility treatment, functional outcome metrics, such as live birth rates (success) and postoperative complications (safety), continue to dominate the area of care assessment, while patient-centeredness is barely addressed. Patient-centered care has been shown to be profitable in the treatment of chronic conditions with a high emotional burden, such as infertility.^[20]^

It is necessary to gain insight into patients’ opinions on infertility treatments. The field of reproductive medicine needs to place greater emphasis not only on “effectiveness” (the percentage of patients who become pregnant) but also on other quality parameters, most notably the degree to which patients are satisfied with the care they receive. By evaluating patients’ perspectives, healthcare providers can gain a deeper understanding of their patients’ desires, requirements, and preferences regarding fertility services and amenities.^[21]^ Interviews and questionnaires are commonly used to measure the satisfaction of patients with their care.^[22–25]^ This is especially crucial if doctors’ and nurses’ impressions of their patients’ experiences with treatment do not properly represent the real situation,^[26]^ as this could hinder their opportunity to make adjustments that are advantageous to their patients.^[27,28]^

The purposes of this study were to identify factors that contribute to patients’ overall satisfaction with IVF clinics in North Cyprus, explore background differences (age, education level, marital status, income, and region of residence) in the evaluation of patients’ quality-of-life (QoL) and satisfaction with infertility services received overseas in North Cyprus, and determine factors that are most likely to affect a patient’s overall experience.

## 2. Materials and methods

### 2.1. Instrument development and measurement

The current study was designed to explore the dimensions of IVF patients’ satisfaction and QoL. Consequently, 2 different surveys were used to study attitudes toward satisfaction and QoL. To study IVF patient satisfaction, qualitative research was used to design a comprehensive questionnaire based on a review of the extant literature. In-depth interviews were conducted with professionals. A total of 25 face-to-face interview invitations were sent to a group of informants comprising specialists and patients from IVF centers in North Cyprus. Of the 25 contacted, 14 agreed to participate. The aim of this phase was to develop a questionnaire to assess the satisfaction of IVF patients. Thus, potential subjects were selected from the IVF process and interviewed based on their experiences, so as to create questions that were clear to the interviewees to enhance their contribution to the interview. Personal interviews with the selected subjects were conducted using open-ended questions. Following a standard method,^[29]^ each interview was directed, recorded, and transcribed. The average duration of the interviews was 45 minutes.

To identify the main effective factors, a qualitative Delphi approach, in addition to thematic coding and analysis, was applied to identify the most influential items. Thus, as a result of the qualitative study, a questionnaire was designed to capture the data based on the objectives and levels of the data to be collected. To modify and confirm the questionnaire, the following 3 groups of experts were asked to participate: 2 professors who majored in healthcare system management; 3 doctors with relevant work experience in IVF centers; and 5 nurses who were aware of IVF systems. These experts confirmed the content validity of measuring the concepts and offered few suggestions for modifications. Ultimately, 69 valid questions remained in the questionnaire (see Appendix 1, Supplemental Digital Content, http://links.lww.com/MD/N211). The results showed that the item, “Did you go to the doctor at the appointed time?” had the lowest average among the items, and the highest average was for the item, “General information such as the foundation year of all IVF centers, the number of IVF patients received, and the number of successful businesses should be given.”

For the 2nd part of this research, which studied the QoL of IVF patients receiving this service, the standard 24 one-option and multiple-choice questionnaire, the fertility QoL questionnaire developed by Cardiff University in 2008, was implemented (see Appendix 2, Supplemental Digital Content, http://links.lww.com/MD/N211). Considering these results, the item with the lowest average was “Has your social life been restricted due to your fertility problems?” The item with the highest average was “Do thoughts of infertility negatively affect your attention and concentration?” All items in both questionnaires were attitudinal measures that were evaluated on a 5-point Likert scale (1 = strongly disagree, 5 = strongly agree).

### 2.2. Sampling and data collection

After confirming the validity of the survey instrument items, the surveys were conducted in the 2nd phase of the study with the same group of respondents to gather data. The survey included 2 questionnaires on IVF patient satisfaction and QoL. The instruments also included demographic questions, such as age, education level, marital status, perceived income adequacy, and region of residence. Fertility levels and policies on fertility differ in different countries; therefore, it is important to classify the responses of patients according to their region of residence when studying issues related to fertility.

Convenience sampling was adopted in this study. The data were collected between February and October 2020. This study recruited IVF patient service users in some IVF clinics in North Cyprus because it has recently become a new and popular medical tourist destination, and, annually, a considerable number of people travel to North Cyprus to receive IVF services. As a result, 145 women were contacted by the centers, and 126 responded. A total of 101 properly filled out questionnaires were retrieved. The respondents voluntarily participated in the survey, and consent forms were collected from all the participants before they started to answer the questionnaire. For the data collection process, informed consent was obtained from all the subjects involved in the study. Furthermore, the study was conducted in accordance with the Declaration of Helsinki and approved by the European University of Lefke Ethics Committee.

### 2.3. Data analysis

Statistical analyses were performed using IBM SPSS 25.0 and R statistical packages. The dataset was analyzed using descriptive statistics to summarize and describe the characteristics of the data. The reliability of the variables was tested by examining the Cronbach alpha coefficient of each variable. For dimension reduction and grouping of the relevant items in the questionnaire and, ultimately, the extraction of the main factors, exploratory factor analysis was employed. Factor analysis is a statistical method used to extract combined factors from several variables. Through this method, it is possible to minimize the number of central variables, which can still represent all the necessary variable information.^[30]^ In this study, principal component analysis was employed, and factors with eigenvalues >1 were extracted. The variable values were obtained by rotating the factor matrix using the varimax method. To indicate the differences between the means of categorical variables in the demographic part of the questionnaires, parametric and nonparametric tests were used. Therefore, initially, a normality test was conducted to determine the type of nonparametric or parametric test. Accordingly, the following tests were selected to indicate the differences in the group means: For normally distributed sample data and categorical variables with 2 groups, an independent t-test was decided. For nonnormally distributed sample data and categorical variables within 2 groups, the Mann–Whitney *U* test was used. For normally distributed sample data and categorical variables with more than 2 groups, a one-way analysis of variance was performed. For nonnormally distributed sample data and categorical variables with more than 2 groups, the Kruskal–Wallis *H* test was used. All dimensions of both questionnaires, which were extracted from the exploratory factor analysis, were investigated based on the demographic categorical variables.

## 3. Results

Table 1 provides details of the participants’ demographic characteristics (see Table 1, which illustrates the demographic characteristics of the participants). A total of 101 patients participated in the survey. About half of the study participants were under 35 years of age (49.5%, n = 50), and the other half were 35 years and above (50.5%, n = 51). Moreover, the majority of the participants were from Western and Eastern European regions (67.3%, n = 68). The majority of the participants were university graduates (77.2%, n = 78). The majority of the participants were married (81.2%, n = 82), and 18.8% (n = 19) were single. Of the participants, 77.3% (n = 78) evaluated their monthly income as average, 16.8% (n = 17) reported low income, and 5.9% (n = 6) had a high income level.

**Table 1:** Demographic findings of the individuals participating in the research*.*

Variables	Category	Frequency	Percentage (%)
Age	Below 35	50	49.5
	35 and above	51	50.5
Region of residence	Central, South and Western Asia	8	7.9
	Europe and Eastern Europe	68	67.3
	Middle East	12	11.9
	North Africa and Africa	8	7.9
	North America	5	5
Level of education	College	17	16.8
	High school	6	5.9
	University	78	77.2
Marital status	Married	82	81.2
	Single	19	18.8
Level of income	Low	17	16.8
	Average	78	77.3
	High	6	5.9

Table 2 shows the reliability analysis results for the subdimensions of the patient satisfaction scale in IVF services with an explanation of the related factors (see Table 2, which shows the dimensions of patient satisfaction scale in IVF services and reliability analysis results). Based on these results, the 1st factor was labeled as “IVF service and mobile application request,” the 2nd factor as “clinical staff’s professionalism,” the 3rd factor was “IVF Service Introduction,” the 4th factor was “medical treatment and transportation,” the 5th factor was “doctor/staff care,” the 6th factor was “IVF counseling service,” and the 7th factor was “doctors’ professionalism.” Moreover, the alpha coefficients for each factor were acceptable (Cronbach alpha > .70), and all were considered reliable.^[31]^

**Table 2:** Dimensions of patient satisfaction scale in IVF services and reliability analysis results.

Factor	Explanation	N	Cronbach alpha
F1	IVF service and mobile application request	6	0.704
F2	Clinical staff professionalism	3	0.802
F3	IVF service introduction	2	0.807
F4	Medical treatment and transportation	3	0.816
F5	Doctor/staff care	3	0.785
F6	IVF counseling service	2	0.744
F7	Doctor professionalism	2	0.732

IVF = in vitro fertilization.

Table 3 shows the reliability analysis results for the subdimensions of the QoL scale for IVF patients with an explanation of the related factors (see Table 3, which demonstrates the dimensions of QoL scale in IVF services and reliability analysis results). These factors were constructed as “emotional,” “mind–body,” “social,” and “relational,” and the reliability results showed that all 4 factors were reliable, since the alpha values were >0.7. The overall reliability of the QoL scale was 0.852, indicating that the scale was reliable for the target population.

**Table 3:** Dimensions of quality-of-life scale in IVF services and reliability analysis results.

Factor	Explanation	N	Cronbach alpha
F1	Emotional	6	0.733
F2	Mind body	6	0.782
F3	Social	6	0.863
F4	Relational	6	0.884

IVF = in vitro fertilization.

Table 4 reports the comparative outcomes of patient satisfaction dimensions based on the categorical variables in this study (see Table 4, which indicates the comparative results of the dimensions of patient satisfaction based on the demographic characteristics of participants). There was a statistically significant difference between IVF service introduction (*P* value = .044 < .05) according to the age groups of individuals in those aged < 35 years (mean rank [MR] = 46.56, standard deviation [SD] = 0.91) versus those aged 35 and above (MR = 55.53, SD = 0.88). Doctors’ professionalism scores of individuals aged 35 years and above (MR = 59.30, SD = 0.74) were significantly (*P *= .003 < .01) higher than those of individuals aged < 35 years (MR = 42.87, SD = 0.85). Clinical staff’s professionalism scores were significantly (*P *= .005 < .01) different in patients from different regions of residence, with the highest score recorded for those from central, south, and western Asia (MR = 75.6, SD = 2.10). Those with university-level education had significantly greater clinical staff’s professionalism scores (*P* = .001 < .01) compared to those with lower education levels (MR = 61.77, SD = 2.21). IVF service and mobile application requests (MR = 53.92, SD = 1.78, *P* = .035 < .01) and doctors’ professionalism scores (MR = 54.81, SD = 0.81, *P *= .004 < .01) were greater in married participants. The clinical staff’s professionalism (*P* *=* .009 < .01) and doctors’ professionalism (*P* = .036 < .01) categories differed significantly in terms of the income level of participants. The clinical staff professionalism scores were highest in those with higher income levels (MR = 61.33 and SD = 2.99). Doctor professionalism scores were highest in those with the lowest level of income (MR = 56.58 and SD = 0.75).

**Table 4:** Comparative results of the dimensions of patient satisfaction based on the demographic characteristics of participants.

Variable	Category	IVF service and mobile application request	Clinical staff professionalism	IVF service introduction	Medical treatment and transportation	Doctor/staff care<?Note To TS: Char = Mixed?>	IVF counseling service	Doctor professionalism
		Mean rank (SD)	Mean rank (SD)	Mean rank (SD)	Mean rank (SD)	Mean rank (SD)	Mean rank (SD)	Mean rank (SD)
Age	Below 35	50.85 (1.73)	46.84 (2.30)	46.56 (0.91)	49.31 (2.09)	48.84 (1.27)	51.29 (1.17)	42.87 (0.85)
	35 and above	51.15 (2.14)	54.02 (2.60)	55.53 (0.88)	52.66 (1.55)	53.12 (1.38)	50.72 (1.41)	59.30 (0.74)
	*P value*	.962	.206	**.044**	.558	.453	.92	**.003**
Region of residence	Central, South and Western Asia	53.50 (1.85)	75.6 (2.10)	57.56 (0.71)	45.75 (0.03)	63.44 (1.36)	30.25 (1.67)	64.06 (0.74)
	Europe and Eastern Europe	50.67 (1.96)	51.40 (2.33)	47.13 (0.91)	52.02 (1.71)	49.13 (1.38)	53.54 (1.22)	47.96 (0.86)
	Middle East	48.04 (2.08)	33.95 (2.27)	58.75 (1.16)	54.04 (2.44)	50.54 (1.14)	52.83 (1.41)	44.917 (0.718)
	North Africa and Africa	60.75 (2.05)	58.69 (2.55)	57.56 (0.71)	48.69 (2.49)	65.44 (1.06)	57.06 (1.04)	59.500 (0.71)
	North America	43.00 (1.79)	25.80 (2.17)	64.00 (0.00)	41.90 (2.19)	34.50 (1.00)	35.60 (0.89)	72.50 (0.89)
	*P value*	.828	**.005**	.161	.902	.236	.132	.134
Level of education	High school	64.33 (0.75)	13.08 (1.23)	55.42 (0.82)	60.17 (1.72)	54.75 (1.47)	42.33 (1.03)	54.75 (1.27)
	College	50.29 (1.57)	50.93 (2.35)	46.56 (0.93)	40.94 (2.18)	46.71 (1.18)	38.88 (1.23)	61.09 (0.69)
	University	50.13 (2.06)	61.77 (2.21)	51.53 (0.91)	52.49 (1.74)	51.65 (1.35)	54.31 (1.30)	48.51 (0.83)
	*P value*	.507	**.001**	.616	.23	.767	.083	.22
Marital status	Married	53.92 (1.78)	51.03 (2.50)	52.23 (0.89)	51.19 (1.90)	50.80 (1.32)	49.63 (1.33)	54.81 (0.81)
	Single	38.42 (2.28)	48.26 (2.32)	45.68 (2.32)	50.18 (1.59)	51.87 (1.36)	56.90 (1.08)	34.58 (0.77)
	*P value*	**.035**	.705	.252	.894	.886	.304	**.004**
Level of income	Low	43.97 (2.34)	48.58 (2.43)	55.45 (0.69)	39.59 (1.44)	58.62 (1.50)	51.91 (0.97)	56.58 (0.75)
	Average	52.34 (1.88)	55.38 (2.43)	49.65 (0.95)	52.18 (1.94)	49.20 (1.31)	50.80 (1.34)	50.88 (0.79)
	High	53.50 (1.38)	61.33 (2.99)	55.42 (0.82)	68.00 (1.17)	52.83 (0.98)	51.00 (1.60)	50.60 (0.86)
	*P value*	.543	**.009**	.533	.083	.461	.989	**.036**

Significant *P* values are indicated in bold.

IVF = in vitro fertilization, SD = standard deviation.

Table 5 illustrates the comparative results of the dimensions of the QoL scale based on the demographic characteristics of the participants (see Table 5, which shows the comparative results of the dimensions of QoL scale based on the demographic characteristics of participants). There were statistically significant differences for the emotional (*P* = .031 < .05), mind-body (*P* = .004 < .01), and relational (*P* = .022 < .05) scores according to the age groups of the participants, with greater emotional scores of individuals aged 35 and above (MR = 53.61, SD = 3.21) than those under 35 (MR = 47.51, SD = 3.25). Higher mind-body scores were recorded for individuals aged 35 and above (MR = 52.70, SD = 4.08) than those under 35 (MR = 49.33, SD = 3.98), and relational scores were higher in those under 35 years (MR = 50.29, SD = 2.50) compared to those aged 35 and above (MR = 44.82, SD = 2.32). The emotional scores of the respondents differed significantly (*P* = .049 < .05) among those from different regions of residence, with greater emotional scores recorded for those from central, southern, and western Asia compared to those from other regions (MR = 73.50, SD = 2.48). Relational scores were significantly different (*P* = .023) and were the highest in those with high income levels (MR = 68.25, SD = 1.47) compared to those with average income (MR = 48.72, SD = 2.40) and low income levels (MR = 34.25, SD = 2.39).

**Table 5:** Comparative results of the dimensions of quality-of-life scale based on the demographic characteristics of participants.

		Emotional	Mind body	Social	Relational
Variables	Category	Mean rank (SD)	Mean rank (SD)	Mean rank (SD)	Mean rank (SD)
Age	Below 35	47.51 (3.25)	49.33 (3.98)	53.75 (3.07)	50.29 (2.50)
	35 and above	53.61 (3.21)	52.70 (4.08)	48.30 (3.05)	44.82 (2.32)
	*P value*	**.031**	**.004**	.267	**.022**
Region of residence	Central, South and Western Asia	73.50 (2.48)	59.81 (2.98)	42.44 (2.26)	39.79 (3.08)
	Europe and Eastern Europe	50.02 (2.92)	50.95 (3.81)	48.99 (3.08)	49.23 (2.44)
	Middle East	45.33 (3.75)	48.292 (5.26)	53.625 (3.39)	46.136 (1.79)
	North Africa and Africa	34.938 (2.97)	41.06 (4.75)	63.50 (3.25)	40.50 (2.17)
	North America	59.25 (5.92)	60.00 (4.06)	65.70 (2.92)	50.70 (3.13)
	*P value*	**.049**	.484	.455	.843
Level of Education	High school	46.50 (4.04)	37.25 (2.32)	42.17 (3.72)	28.25 (2.32)
	College	49.47 (2.86)	49.21 (3.10)	47.19 (2.61)	42.79 (2.55)
	University	50.98 (3.29)	52.45 (4.30)	52.53 (3.13)	50.25 (2.35)
	*P value*	.932	.452	.587	.144
Marital Status	Married	51.35 (3.43)	49.99 (4.20)	50.07 (3.01)	46.76 (2.39)
	Single	46.87 (2.22)	55.37 (3.19)	55.03 (3.33)	50.64 (2.53)
	*P value*	.544	.541	.37	.575
Level of Income	Low	53.60 (3.12)	51.53 (3.90)	50.51 (3.01)	34.25 (2.39)
	Average	51.58 (3.67)	67.75 (4.15)	74.17 (1.23)	48.72 (2.40)
	High	36.06 (3.24)	42.68 (4.44)	45.09 (3.47)	68.25 (1.47)
	*P value*	.065	.256	.105	**.023**

Significant *P* values are indicated in bold.

SD = standard deviation.

### 3.1. Pandemic versus post-pandemic

The findings presented in Table 4 indicate that certain dimensions of patient satisfaction, such as IVF service and mobile application requests, clinical staff’s professionalism, IVF service introduction, and doctors’ professionalism, were significantly influenced by the demographic characteristics of the participants (age, region of residence, level of education, marital status, and level of income) during the COVID-19 pandemic. By contrast, dimensions such as medical treatment and transportation, doctor/staff care, and IVF counseling service were not significant. Regarding the demographic characteristics of the participants (such as age, region of residence, level of education, marital status, and level of income), certain dimensions of the QoL scale were found to be statistically significant. Specifically, the emotional, mind-body, and relational dimensions of the QoL scale were found to be significant, while the social dimension was not significant.

To confirm whether the explored significant dimensions remained important in the post-pandemic era, we conducted a qualitative study. We conducted 12 in-depth interviews with experts and staff members, including doctors, nurses, and coordinators from different IVF centers to ensure that the responses were professional and reliable. The dimensions were ranked according to the interviewees’ opinions. Table 6 indicates that 8 out of the 12 interviewees (66.66%) highlighted the importance of IVF counseling services, whereas 7 interviewees (58.33%) emphasized the importance of the doctor/staff care dimension (See Table 6, which illustrates the post-pandemic interview results for patient satisfaction dimensions). Furthermore, half of the participants (50%) emphasized the importance of the IVF service and mobile application request dimension. Conversely, the IVF service introduction dimension is considered the most crucial by only 3 interviewees, accounting for 25%. Robertson et al^[32]^ highlighted the importance of IVF counseling services after the COVID-19 pandemic. A comparison of noteworthy dimensions of patient satisfaction during the pandemic (Table 4) with the insights gathered from interviews regarding the importance of the dimensions post-pandemic (Table 6) revealed that the IVF service introduction dimension held significance during the pandemic. Nevertheless, in the post-pandemic era, its significance diminished, according to the perspectives of the interviewees, who prioritized patient care as a far more crucial matter. Moreover, the medical treatment and transportation dimension was deemed negligible during the pandemic but gained importance after the pandemic. According to the respondents, patient transfers were a complex issue during the pandemic. Since then, the importance of patient transfers has increased for both patients and IVF centers. Furthermore, the importance of the doctor/staff care dimension was negligible during the pandemic, but it became exceedingly crucial after the pandemic. According to the respondents, patient satisfaction after the pandemic was heavily influenced by the quality of care provided to patients. Furthermore, the importance of the IVF counseling service dimension diminished during the pandemic, but it emerged as the most crucial dimension of patient satisfaction after the pandemic. The rationale for this is that the pandemic significantly heightened the stress levels connected with IVF, making mental assistance indispensable for patients post-pandemic. The IVF service and mobile application request, clinical staffs’ professionalism, and doctors’ professionalism characteristics maintained substantial importance both during and after the pandemic.

**Table 6:** Post-pandemic interview results for patient satisfaction dimensions.

Dimensions	Frequency	Percentage
IVF service and mobile application request	6	50
Clinical staff professionalism	5	41.66
IVF service introduction	3	25
Medical treatment and transportation	5	41.66
Doctor/staff care	7	58.33
IVF counseling service	8	66.66
Doctor professionalism	5	41.66

IVF = in vitro fertilization.

Similarly, the interviewees ranked the dimensions of the QoL scale in terms of their importance for the post-pandemic era, ranging from the highest level of importance to the lowest. According to the rankings given by the interviewees (see Table 7, which demonstrates the post-pandemic interview results for QoL scale dimensions), the Emotional dimension of the QoL scale was deemed the most significant by 11 interviewees (91.66%), followed by the mind body dimension (6 interviewees, 50%), social dimension (5 interviewees, 41.66%), and relational dimension (2 interviewees, 16.66%). Amid the pandemic, the results suggested that the social dimension of the QoL scale was the sole dimension that lacked significance. However, the rating of the interviewees suggested that it became the third-most important dimension of the QoL scale post-pandemic. Interviewees perceived that due to the isolation caused by the pandemic, having social support and social interactions became vital for patients after the pandemic.

**Table 7:** Post-pandemic interview results for quality-of-life scale dimensions.

Dimensions	Frequency	Percentage
Emotional	11	91.66
Mind body	6	50
Social	5	41.66
Relational	2	16.66

## 4. Discussion

The results of this study indicate that age is a substantial antecedent of patient satisfaction with the introduction of IVF services and doctors’ professionalism. Previous research has shown conflicting results, considering the nature of the relationship between patient age and satisfaction.^[33,34]^ The findings of this study showed that the average degree of patient satisfaction with the introduction of IVF services improved with patient age, with the highest levels of satisfaction reported by patients who were 35 years old. Similarly, doctors’ professionalism was another significant factor for greater satisfaction among older patients than among younger patients who used IVF treatment. Lack of experience (and perhaps unreasonable demands) with healthcare institutions may account for younger individuals’ lower satisfaction ratings.^[35,36]^

Patients who had a higher level of education reported a higher level of satisfaction in terms of clinical staff’s professionalism compared to patients who had a lower level of education. This indicates that the mean level of satisfaction with staff skills was higher in patients with a higher level of education. One argument could be that people’s critical thinking and expectations increase in parallel with their level of education. As this study found, a higher level of education may lead to a more critical assessment of the professionalism of medical professionals based on the individual’s knowledge and preferences, which is consistent with prior research^[37–44]^ showing that higher socioeconomic status and education levels are associated with higher levels of satisfaction with fertility treatment among both men and women. The patients’ marital status was also found to be associated with their overall satisfaction level in terms of IVF service, mobile application requests, and doctors’ professionalism. Overall, the results of this study are consistent with those of earlier research showing that single patients were less satisfied with IVF services than married patients.^[45,46]^ To provide better treatment centered on the patient, those who provide medical services should look for ways to get patients more involved in and concerned about their health. It is possible that using applications designed to run on smart devices could be a method to boost patient involvement in medical services. Applications have been used to educate patients about ways to improve their health, involve them in monitoring illness treatment, and assist them in adhering to treatment regimens.

Based on the findings of this study, lower-income and lower-educated patients were less satisfied with their IVF treatments. For example, they were less likely to be satisfied with the professionalism of doctors and clinical staff, which is in line with the findings of previous studies.^[47,48]^ Assuming that customer satisfaction is the result of a positive evaluation of a product or service after purchase could provide some insight into this issue.^[49]^ The level of customer satisfaction can be determined by contrasting the customer’s expectations with the actual results; if the actual performance is superior, the customer is satisfied. The phenomenon of perceptions being greater than actual achievement is the root cause of dissatisfaction.^[50]^ Customer satisfaction is affected by their perception of service quality.^[51]^ Thus, people with higher educational levels and incomes who used IVF services in North Cyprus expected doctors’ and clinical staff’s professionalism to be outstanding.

In the current study, patients from different regions of residence reported significantly different levels of satisfaction with the professionalism of clinical staff. People from central, southern, and western Asia had the highest levels of satisfaction compared to individuals from other regions. Studies have shown that there are geographical and cultural differences in fertility levels and approaches to fertility treatment.^[52]^ Therefore, people from other cultures have varying cultural norms, expectations, and experiences with fertility services, which impact their satisfaction with IVF treatment.^[53]^ Furthermore, the findings from the interviews indicate that IVF counseling services and the doctor and staff care have become exceptionally crucial in the post-pandemic era for IVF services. This is due to the increased demand for emotional and psychological support among patients who have already experienced the stressful period of the pandemic and may encounter additional stress when seeking IVF services. In addition, the quality of care provided by the doctor and staff is crucial since it directly impacts patient happiness and the overall effectiveness of the treatment.

A secondary objective of this study was to investigate the QoL and demographic characteristics of patients receiving IVF in North Cyprus. According to the findings of the study, a relatively young age is a determinant of a lower score in the emotional and mind–body domains of QoL. By contrast, older patients ranked lower in the relational subcategory of QoL. The importance of childbearing among infertile older couples could likely have a detrimental impact on their relationships. This could explain the link between being older and having a worse relational QoL domain score. These results are comparable to those reported in previous investigations.^[54,55]^ The results of this study further showed that living in different regions is a potential indicator of QoL in the emotional subdimension. The patients from central, southern, and western Asia; North America, Europe, and Eastern Europe; the Middle East; and North Africa and Africa recorded the highest to lowest levels of emotional scores, respectively. This could be due to the fact that people come from different cultural backgrounds and have different expectations of society, both of which contribute to the stigma associated with infertility issues and childlessness. Additionally, the findings from the interviews indicate that the emotional aspect of QoL is crucial in post-pandemic era. This is because the emotional well-being of patients holds significant importance, as they may encounter stress and anxiety when seeking treatment, in addition to the overall increased stress caused by the pandemic.

### 4.1. Limitations and future studies

The first limitation of this study is that not every relevant variable could have been included in the analysis. Therefore, generalization difficulties may arise owing to the influence of the unknown parameters. For instance, patients were selected from a variety of IVF clinics; therefore, differences in care providers were not considered. This should be considered in future research. Second, based on the nature of IVF services, most centers are unwilling to help researchers with their surveys because of confidentiality issues, which hinder accessing patients and collecting data. Third, as the data were collected during the COVID-19 pandemic, the number of patients was considerably lower because of travel restrictions. Fourth, one of the main difficulties in conducting this type of research is accessing patients through IVF centers. This reverts to the nature of this service, which is mostly a very confidential process because of the patient’s characteristics, which is also the main aim of this study. People are still guarded about revealing their personal information; thus, IVF centers keep their information very private. To overcome this difficulty, the authors leveraged their practical experiences in the field of IVF and applied a very professional and confidential approach to seek permission from some IVF clinics to contact some of their patients to participate in this research.

### 4.2. Conclusions

The results of this study have significant implications for both routine procedures performed in IVF facilities and future research. All reproductive clinics can use the insights gained from this study to determine and prioritize patient-centered improvement activities and focus on quality enhancement. This research suggests that average fertility clinics should place a premium on addressing patient needs. Efforts should be directed toward creating more complex models to explicate patient satisfaction findings. People attribute high levels of technical skills to an expert if the treatment they receive from that specialist is provided in a pleasant and knowledgeable manner. It can be concluded that patients undergoing infertility treatment may be quite flexible and welcome numerous treatments if they have confidence in a particular doctor’s ability to properly treat them. Clinicians and IVF center personnel need to be sensitive to the needs and desires of their patients, considering each person’s background and level of comprehension of the operations they will undergo.

## Author contributions

**Conceptualization:** Naskhanym Chausheva.

**Data curation:** Naskhanym Chausheva.

**Formal analysis:** Naskhanym Chausheva.

**Investigation:** Naskhanym Chausheva, Macide Artac Ozdal.

**Methodology:** Naskhanym Chausheva, Macide Artac Ozdal.

**Resources:** Naskhanym Chausheva.

**Software:** Naskhanym Chausheva.

**Supervision:** Macide Artac Ozdal.

**Validation:** Macide Artac Ozdal.

**Visualization:** Macide Artac Ozdal.

**Writing – original draft:** Naskhanym Chausheva.

**Writing – review & editing:** Macide Artac Ozdal.

## Supplementary Material


