# Supplementary material for: The impact of IVF patients’ characteristics on their satisfaction and quality-of-life with overseas treatment: A mixed methods approach
Source: Medicine (Baltimore). 2024 Jul 19;103(29):e38682. doi: 10.1097/MD.0000000000038682 (PMC11398773; doi:10.1097/MD.0000000000038682)
Supplement: Supplementary file 1 [file medi-103-e38682-s001.pdf]

EUROPEAN UNIVERSITY OF LEFKE
University Ethics Committee (UEC)

ETHICS COMMITTEE REPORT

**Ethical Review Subject:**	Thesis entitled "Evaluation of Management of In Vitro Fertilization (IVF) services in terms of Medical Tourism in Turkish Republic of Northern Cyprus" written by PhD student Naskhanym Chausheva with student ID 164224 – Department of Health Management, Institute of Graduate Studies and Research
**Date the subject came to the Ethics Committee:**	30.07.2019
**Date the subject was reviewed in the Ethics Committee:**	18.08.2019
**Ethics Committee Decision Date and Number:**	10.09.2019, ÜEK/43/01/09/1920/03

DECISION

1.	[√] Accept
2.	[ ] Revision is required:<br>There are questions/items, processes or elements that may include ethical issues.<br>Explanation:
3.	[ ] Reject<br>Justification, Opinion, Suggestion and Explanations:

On behalf of University Ethics Committee (UEC) members who are present to discuss the ethics review and have no direct or indirect relationship to the subject

Prof. Dr. Hüseyin Oğuz
Chairman
